# Association of *IL-1B* rs16944 Polymorphism With Acute Encephalopathy With Biphasic Seizures and Late Reduced Diffusion Is Opposite to That of Febrile Seizures

**DOI:** 10.3389/fneur.2022.891721

**Published:** 2022-05-30

**Authors:** Akiko Shibata, Mariko Kasai, Ai Hoshino, Masashi Mizuguchi

**Affiliations:** ^1^Department of Developmental Medical Sciences, Graduate School of Medicine, The University of Tokyo, Tokyo, Japan; ^2^Department of Pediatrics, Graduate School of Medicine, The University of Tokyo, Tokyo, Japan; ^3^Department of Pediatrics, National Rehabilitation Center for Children With Disabilities, Tokyo, Japan

**Keywords:** *IL-1B*-511, acute encephalopathy with biphasic seizures and late reduced diffusion (AESD), status epilepticus, association study, rs16944, genetic risk factor

## Abstract

**Objective:**

Acute encephalopathy with biphasic seizures and late reduced diffusion (AESD) is a severe neurologic complication of febrile infectious diseases in children. At the onset, AESD is clinically manifested as febrile status epilepticus. Subsequent damage to the cerebral cortex is ascribed to neurotoxicity. The incidence of AESD is remarkably high in Japan, suggesting the involvement of genetic factors. The expression of interleukin 1 beta (IL-1β), a member of the cytokine family involved in the inflammatory response, is reportedly associated with rs16944, a polymorphism in the upstream region of the *IL-1B* gene, being higher in TT genotype. Previous association studies of rs16944 with febrile seizures (FS) have demonstrated a significant excess in the TT vs. CC + CT genotype in the Asian population. Here, we conducted a case-control association study of rs16944 in AESD.

**Methods:**

We genotyped rs16944 by Sanger sequencing on 283 patients with AESD. As controls, we used genotyping data of 104 Japanese individuals obtained from the 1,000 Genomes Project. Then, we performed a case-control association study using the chi-square test.

**Results:**

The ratio of individuals with TT vs. those with CC+CT genotype was significantly lower in AESD than in the controls [*p*-value 0.021, Odds Ratio (OR) 0.52]. This finding was opposite to that of a previously reported FS.

**Conclusion:**

The AESD has a genetic background distinct from FS and is not a severe type of FS.

## Introduction

Acute encephalopathy with biphasic seizures and late reduced diffusion (AESD) is a rare and intractable neurologic disorder severely affecting the cerebral cortex of infants and young children. The initial or early seizure of AESD is usually a prolonged generalized convulsion with high fever due to common infectious diseases, such as influenza and exanthem subitum. Several days later, the second or late seizure appears as a cluster of focal seizures, followed by signs of cerebral cortical dysfunction and intractable epilepsy. The mechanism of cerebral cortical damage is considered to be excitotoxic neuronal death triggered by febrile status epilepticus (1). The incidence of AESD is remarkably high in East Asia, especially in Japan (2, 3), suggesting the involvement of genetic factors in AESD.

Febrile seizures (FS), on the other hand, are the most common condition of seizures in children. The prevalence of FS is higher in Japan (6–9%) than in European and North American countries (2–5%) (4). Simple FS are clinically benign, whereas complex FS and febrile status epilepticus may produce neurological sequelae and/or predispose to later epilepsies including temporal lobe epilepsy. Moreover, it is often clinically difficult to distinguish between prolonged FS and initial seizures of AESD.

Increasing evidence supports the involvement of inflammatory processes in the pathogenesis of seizures of different etiologies, such as infection, fever, neurotrauma, and stroke (5–7). Several studies have focused on the proinflammatory cytokine interleukin-1β (IL-1β) and its role in FS (7–9). There is an upstream variant, rs16944 (*IL-1B*−511 T>C, NG_008851.1:g.4490T>C), in the promotor of *IL-1B*. According to ALFA (Allele Frequency Aggregator), T allele frequency in total, European, and East Asian populations is 0.357, 0.335, and 0.477, respectively (www.ncbi.nlm.nih.gov/snp/docs/gsr/alfa/). Expression of IL-1β is highest in TT genotype and lowest in CC (10). The frequencies of the T allele and TT homozygotes are significantly higher in Japanese children with simple FS than in controls (11). A meta-analysis of association studies of rs16944 with FS reported a significant excess in the TT vs. CC + CT genotype in the Asian population (8). However, no research has explored the association between rs16944 and AESD.

In this study, to explore whether AESD and FS share a common genetic background, we investigated the association between the *IL-1B* polymorphism rs16944 and AESD.

## Materials and Methods

### Cases

We recruited Japanese patients with AESD from hospitals in Japan from the year 2008 to 2018, based on the diagnostic criteria consisting of characteristic clinical course, biphasic seizures, typical MRI findings, and delayed appearance of the cerebral subcortical white matter lesions (2, 3). In this study, we adopted the same inclusion criteria as those in our previous study; we included patients with “definite” AESD, fulfilling both the clinical and MRI criteria, and those with “probable” AESD meeting of either of them (12). The patients without late seizures had super refractory or prolonged initial seizures and were mechanically ventilated under high dose barbiturate. Consequently, they had no clinically apparent late seizures. The patients without MRI findings were also critically ill and were intubated, so it was difficult to obtain MR images at the appropriate timing for ADC changes. Therefore, we included “probable” patients with AESD as clinically conceived patients with AESD.

A total of 283 Japanese patients with AESD were enrolled in this study. All patients were Japanese and mutually unrelated. The clinical characteristics of the patients are shown in **Table 2**.

This study was reviewed and approved by the Institutional Review Board of the University of Tokyo (No. G3504). We obtained written informed consent from the parents of the patients.

### Control

As controls, we used genotyping data of Japanese populations (JPT) obtained from the 1000 Genomes Project. JPT individuals consist of 104 healthy Japanese adults (13).

### Genetic Analysis

Peripheral blood samples were collected from the patients. Genomic DNA extraction and polymerase chain reaction (PCR) was conducted using a standard protocol. The GRCh37 consensus genome sequence was used as a reference genome (14). A total of 304 base-pair genomic regions, including rs16944, were amplified with primers described in the previous study (Table 1) (15). PCR amplification was performed using AmpliTaq PCR kits (Applied Biosystems). The reaction mixture contained 2 μl buffer, 2 μl of 2 mM dNTP, 1 μl forward and reverse primers (10 pmol), 0.12 μl AmpliTaq, and 1 μl genomic DNA (30 ng). All the PCR products were purified with a PCR product sequencing kit (Amersham Biosciences, Little Chalfont, Buckinghamshire, UK), and were reacted with the Big Dye Terminator FS ready-reaction kit (Applied Biosystems, Foster City, CA, USA). Purified PCR products were sequenced on 310 Genetic Analyzer, 3100 Genetic Analyzer, or 3130xl Genetic Analyzer (Life Technologies, Carlsbad, CA, USA).

**Table 1:** Primers and PCR conditions.

**Primers**
Upstream primer	5' - TGGCATTGATCTGGTTCATC - 3'
Downstream primer	5' - GTTTAGGAATCTTCCCACTT - 3'
**PCR conditions**
Denaturation	95°C, 30 s
Annealing	55°C, 30 s
Extension	72°C, 45 s
Number of cycles	34
Product size	304 base pair

### Statistical Analysis

We conducted a case-control association study of rs16944 using the chi-square test. The statistical analysis was conducted using R software (version 3.5.1) (16). A *p*-value <0.05 was considered to indicate a significant difference in the present study.

## Results

### Clinical Characteristics of the Patients

Clinical features of the patients were similar to those in our previous epidemiological survey (2, 3). At the onset of AESD, many of the children (61%) were younger than 24 months. The most common pathogen of preceding infection was human herpesvirus type 6 or 7. History information was available in 269 patients. Twenty-nine patients had simple febrile seizures, 12 had epilepsy, 8 had some forms of encephalopathy, 9 had intellectual disability, and 2 had acute disseminated encephalomyelitides. There were two patients with tuberous sclerosis complex, and one each with α thalassemia X-linked intellectual disability syndrome, chromosomal abnormality, autism spectrum disorder, periventricular leukomalacia, and spinal muscular atrophy. The duration of initial seizures was longer than 15 min in about 82% of the cases. Late seizures were noted in 73%, and subcortical MRI lesions in 90%. With regard to neurological sequelae, we collected information on motor and intellectual deficits. If the two deficits were different in degree, the more severe was adopted for grading. More than 60% of the patients were left with neurological sequelae. Among the 283 patients with information on outcome, deficits were profound in 34 patients (12%), severe in 47 (16.6%), moderate in 32 (11.3%), mild in 51 (18%), and none in 56 (19.8%) (Table 2).

**Table 2:** Clinical characteristics of the patients.

**Clinical features**	**Patients (%) (*N* = 283)**
**Month**	
1-6	8 (2.8)
7-12	61 (21.6)
13-18	69 (24.4)
19-24	36 (12.7)
> 24	106 (37.5)
Unknown	3 (1)
**Sex**	
Female/Male	152 /129 (53.7/45.6)
Unknown	2 (0.7)
**Pathogen**	
Human herpes virus 6/7	90 (31.8)
Influenza virus	32 (11.3)
Respiratory syncytial virus	13 (4.6)
Others	116 (40.6)
Unknown	32 (11.7)
**Duration of initial seizure**	
<15 min	37 (13.1)
>15 min	233 (82.3)
Unknown	13 (4.6)
**Late seizures**	
Positive	208 (73.5)
None	59 (20.8)
None with intubation or sedation	6 (2.1)
Unknown	10 (3.5)
**Distribution of lesions**	
Frontal	47 (16.6)
Hemispheric	48 (17)
Diffuse	102 (36)
Others	59 (20.8)
None	17 (6)
Unknown	10 (3.5)
**Neurological sequelae**	
Profound	34 (12)
Severe	47 (16.6)
Moderate	32 (11.3)
Mild	51 (18)
None	56 (19.8)
Unknown	63 (22.3)

*Intellectual deficit was graded into none, mild (DQ 50–70), moderate (DQ 35–50), severe (DQ 20–35), and profound (DQ <20). Motor deficit was graded into none, mild (able to walk without support), moderate (unable to walk without support), severe (unable to sit), and profound (bedridden). The motor deficit criteria were adjusted according to patients' age and motor development before the onset of acute encephalopathy. The outcome was judged by pediatricians who treated the patients. Overall neurological sequelae were defined according to the grade, which was more severe between motor and intellectual deficits*.

### Case-Control Association Study of rs16944 in AESD

The ratio of TT genotype in the AESD cohort was 14.1%, whereas that in the controls was 24%. The ratio of TT genotype was significantly lower in AESD cases than in controls (*p* = 0.021, OR = 0.52, chi-square test, Table 3).

**Table 3:** Case-control association study of rs16944 in acute encephalopathy with biphasic seizures and late reduced diffusion (AESD).

		**AESD**	**Controls**
Allele frequency	C	0.578	0.534
	T	0.422	0.466
Genotype frequency	CC	0.297	0.308
	CT	0.562	0.452
	TT	0.141	0.24
HWE			
*p*-value		0.011	0.35
Chi-square test (TT vs CC+CT)			
*p*-value		0.021	
Odds ratio (95%CI)		0.52 (0.297-0.910)	

*HWE, Hardy Weinberg Equilibrium; 95% CI, 95% confidence interval*.

When we analyzed the association between sex and genotype, TT genotype was significantly higher in females (*p* = 0.03). There was no association between genotype and type of initial seizures or outcomes.

## Discussion

In this study, the ratio of individuals with TT, a higher producer of IL-1β, was significantly lower in AESD than in the controls. This finding was opposite to that previously reported on FS: a significant excess in the TT vs. CC + CT genotype (8, 11). Although we found that the TT genotype was significantly higher in females, we suppose that it might be due to sampling bias. There was no association between rs16944 and the type of initial seizures or outcome.

Clinical and basic studies have demonstrated that hyperactivation of IL-1β plays important role in the pathogenesis of febrile and epileptic seizures. Intrahippocampal injection of IL-1β before focal application of kainic acid doubled the duration of the seizures induced by kainate in animal models by enhancing glutamatergic neurotransmission (17). According to a systematic review and meta-analysis on cytokines in FS, high levels of cerebrospinal fluid IL-1β and serum IL-6 are associated with an increased risk of FSs in children (18). In drug-resistant epilepsy, the levels of IL-1β+ CD14+ monocytes are reportedly correlated with seizure frequency (19).

Several association studies of rs16944 have previously been conducted in FS and temporal lobe epilepsy. As we mentioned before, a meta-analysis of polymorphisms in FS reported a significant excess in the TT vs. CC + CT genotype in the Asian population (8). TT genotype is also overrepresented in Japanese patients with temporal lobe epilepsy with hippocampal sclerosis (9). Taken together, these genetic studies suggested the detrimental role of IL-lβ in the pathogenesis of FS and temporal lobe epilepsy.

To find biomarkers of AESD, inflammatory cytokines in biological samples of patients with AESD have been vigorously investigated. Several researchers have reported a significant association between AESD and blood cytokines, such as IL-6, IL-10, and TNF-α (20, 21). On the other hand, a study in Japan has reported that serum IL-1β was significantly lower in the patients with AESD with HHV-6 infection than in the controls with exanthem subitem (22), which may be relevant to the finding of our study.

In the field of basic neuroscience, the effects of IL-1β on both excitatory and inhibitory neurotransmitters have been reported. IL-1β can participate in the development of seizures and epileptogenesis through the influence on the calcium influx across the N-methyl- D-aspartate (NMDA) glutamate receptor, the reduction of glutamate uptake by astrocytes, and the increase of glutamate release by glial cells (23, 24). As for inhibitory neurons, fast inhibitory neurotransmission in the brain is principally mediated by the neurotransmitter γ-aminobutyric acid (GABA) and its synaptic target, the type A GABA receptor (GABA_A_ receptor) (25). IL-1β enhances cell-surface expression of GABA_A_ receptors, increases GABAergic tone, and alters synaptic strength at central GABAergic synapses, thereby contributing to cognitive dysfunction in sepsis-associated-encephalopathy (26). The critical role of IL-1β/IL-1 receptor signaling in neuroprotection has been demonstrated by an experimental study using an excitotoxin-damaged mouse retina (27).

Our results suggest that AESD has a genetic background that is partially distinct from FS and temporal lobe epilepsy, and that AESD is not simply a severe form of FS. The differences between our findings on AESD and previous findings on FS may suggest the two faces of IL-1β in fever-induced seizures: a risk factor for seizure and hippocampal sclerosis, and a protective factor against excitotoxic damage to the cerebral cortex. At present, the dual nature of IL-1β in a child's brain remains hypothetical and warrants further investigation.

We did not conduct multivariate logistic regression because statistical power seemed weak due to the small sample size. If we can collect sufficient samples in the future, it would be very informative in considering the relationship between *IL-1B* and AESD.

In this study, one-fourth of the AESD cases was “symptomatic” with various underlying neurological disorders, and this is in agreement with the findings of a previous study that noted such disorders in one-third of AESD (28). Many of these disorders are caused by variations in genes other than *IL-1B*. Genetically, AESD is a multifactorial disorder associated with multiple susceptibility genes. Taking these facts into consideration, we decided not to exclude the symptomatic cases from this IL-1B association study.

In our previous genome-wide association study (GWAS) of AESD, rs16944 did not reach genome-wide significance (29). GWAS is a hypothesis-free approach, whereas the present study took a candidate gene approach based on the clinical similarity between AESD and FS, and the association of FS and IL-1β known previously, which we consider can justify the design of this study.

Our results suggest that AESD has a genetic background distinct, at least partially, from FS, and that AESD is not simply a severe form of FS.

## Data Availability Statement

The datasets presented in this study can be found in online repositories. The names of the repository and accession number can be found below: [NBDC Human Database, hum0347.v1.freq.v1, https://humandbs.biosciencedbc.jp/en/hum0347-v1].

## Ethics Statement

The studies involving human participants were reviewed and approved by the Institutional Review Board of the University of Tokyo. Written informed consent to participate in this study was provided by the participants' legal guardian/next of kin.

## Author Contributions

AS, AH, and MM contributed to conception and design of the study. AS performed the statistical analysis and wrote the first draft of the manuscript. All authors contributed to manuscript revision, read, and approved the submitted version.

## Funding

This research was supported by a Grant-in-Aid for Scientific Research, No. 15H04872, from the Japan Society for the Promotion of Science, and a Grant-in-aid for Policy Research for Intractable Diseases, No. H30-Nanji-Ippan-007/21FC1005, from the National Institute of Public Health, Japan.

## Conflict of Interest

The authors declare that the research was conducted in the absence of any commercial or financial relationships that could be construed as a potential conflict of interest.

## Publisher's Note

All claims expressed in this article are solely those of the authors and do not necessarily represent those of their affiliated organizations, or those of the publisher, the editors and the reviewers. Any product that may be evaluated in this article, or claim that may be made by its manufacturer, is not guaranteed or endorsed by the publisher.
